# Attitude of Physicians towards Periodontal Disease and Diabetes Mellitus Screening in Dental Clinics in Thailand

**DOI:** 10.3390/ijerph18105385

**Published:** 2021-05-18

**Authors:** Manatsara Panakhup, Intouch Lertpanomwan, Chayaphat Pajonklaew, Tawepong Arayapisit, Suraphong Yuma, Patr Pujarern, Tharee Champirat, Naiyana Buranachad, Pornpoj Fuangtharnthip, Chanita Tantipoj

**Affiliations:** 1 Department of Advanced General Dentistry, Faculty of Dentistry, Mahidol University, Ratchathewi, Bangkok 10400, Thailand; manatsara.pan@mahidol.ac.th (M.P.); patr.puj@mahidol.ac.th (P.P.); tharee.cha@mahidol.ac.th (T.C.); naiyana.boo@mahidol.ac.th (N.B.); pornpoj.fun@mahidol.ac.th (P.F.); 2 Mahidol International Dental School, Faculty of Dentistry, Mahidol University, Ratchathewi, Bangkok 10400, Thailand; anslertpanomwan@gmail.com (I.L.); chayaphat.paj@gmail.com (C.P.); 3 Department of Anatomy, Faculty of Dentistry, Mahidol University, Ratchathewi, Bangkok 10400, Thailand; tawepong.ara@mahidol.ac.th; 4 Department of Physics, Faculty of Science, Mahidol University, Ratchathewi, Bangkok 10400, Thailand; suraphong.yum@mahidol.ac.th

**Keywords:** attitude, screening, diabetes mellitus, physician, dental clinic, periodontal disease

## Abstract

Background: Diabetes mellitus (DM) is one of the top causes of death in many places of the world. Diagnosing DM in the early stage is necessary to avoid severe cases and death. Objectives: To evaluate the knowledge of association between DM and periodontal disease (PD) among Thai physicians and assess their attitudes towards DM screening in dental clinics in Thailand. Methods: Online survey of currently practicing physicians in Thailand was conducted on voluntary basis using the newly developed questionnaire. Result: We received 403 responses that are statistically sufficient to represent the entire population of currently practicing physicians in Thailand. A total of 97.3% of all responses indicate that Thai physicians have knowledge about the association between DM and PD. More than 90% know that DM has an effect on PD; however, 70% know about the effect of untreated PD in DM patients. Most of physicians think that DM screening in dental clinics is important (79.1%) and are ready to accept referred cases for definite DM diagnosis from a dentist (84.1%). The concerned issues among the participants were the accuracy of the test results in DM screening (73%) and ability of a dentist to perform the screening (71.5%). Conclusions: The majority of participating physicians have adequate knowledge about the bidirectional relationship between DM and PD. They have a positive attitude towards DM screening in dental setting. The collaboration between physicians and dental professionals should be established to reduce the number of undiagnosed DM patients and enhance the medical care of DM patients.

## 1. Introduction

Type 2 Diabetes Mellitus (DM) is one of the most common non-communicable diseases. It is the sixth cause of death and mortality in Thai population. In its early stage, DM does not exhibit any symptoms and may not be diagnosed for many years until it evolves into severe conditions [1]. A substantial proportion of DM cases remains undiagnosed. Many patients are not aware that they have DM and do not receive a diagnosis until having complications including periodontal disease (PD). Greenberg et al., (2007) [2] reported that individuals with a self-reported family history of DM, hypertension, high cholesterol levels, and clinical evidence of PD have undiagnosed DM with a probability of 27% to 53%. Early detection and prompt treatment may help reduce the burden of DM and its complications.

Various studies [1,3,4] reported a bidirectional relationship between DM and PD. Chronic periodontitis is a typical PD in adults. It is a potentially progressive bacterial infection in which inflammatory response plays a major role. Chronic periodontitis may lead to tooth loss caused by extensive destruction of periodontal attachment. The DM patients, who are diagnosed with DM, are considered as a high-risk group with greater susceptibility to severe forms of periodontal breakdown [5]. Clinical studies have shown that subjects with DM have a greater prevalence of periodontal complications than the healthy individuals, in particular in oral cavity [5,6,7]. In addition, DM patients who have poor glycemic control bring unwelcome consequences of periodontal health probably leading to tooth loss [8]. Contrariwise, DM patients with severe periodontitis may have an increasing risk of poor glycemic control [5]. Tantipoj et al., (2017) [9] assessed risk factors of having DM in Thai dental patients. They found the prevalence of 33.8% undiagnosed DM in the dental patients with a hyperglycemic condition (HbA1c ≥ 5.7%). The prevalence is significantly higher than roughly 13% in the normal Thai population. A recent cost–benefit study by Nasseh et al., (2014) [10] estimated that screening for chronic disease conditions in a dental setting could save on the cost of health care. Therefore, the dental clinic can be an important venue to screen for DM in undiagnosed patients.

According to the World Health Organization (WHO, 2005) [11], oral diseases including PD are serious and essential parts in the general health of individuals. There should be more collaboration between dentists and medical doctors to closely monitor both oral and physical health of patients. A large amount of research has demonstrated the efficacy and potential yield of medical screening in a dental setting [2,12,13]. Their purposes are to identify at-risk asymptomatic patients who are unaware of their risks of developing systematic diseases such as cardiovascular disease and DM and who could benefit from strategies to prevent disease onset and control disease severity. Tantipoj et al. [14] developed a questionnaire to explore attitudes of Thai dentists and dental patients towards DM screening in a dental clinic. They revealed that the majority of the participating dentists were willing to screen for DM using a test that yield an immediate result, discuss the result with the patients, and refer the patient who is at risk of having DM to follow up with a doctor. Similarly, most of the dental patients felt that DM screening in the dental clinic was important and they were willing to have a DM screening at a dental clinic [9]. Success in DM screening at a dental setting strongly depends on whether or not medical doctors are willing to accept the referred cases and provide the definite diagnosis of the disease. However, the attitudes of the medical doctors in Thailand are still unknown. This work is the first study to explore the attitudes of medical doctors in Thailand towards the DM screening in a dental clinic. We examined the attitudes of Thai physicians towards the DM screening in a dental clinic in Thailand and investigated their knowledge on the association between DM and PD.

## 2. Materials and Methods

This study was a cross-sectional study conducted between April and November 2019. The participants were physicians who received a Thai medical license certificate and were still practicing medicine in Thailand. This study was approved by the Ethics Committee of Human Research, Faculty of Dentistry and Faculty of Pharmacy, Mahidol University, Bangkok, Thailand (No.MU-DT/PY-IRB 2019/005.2501).

We determined the sample size of our study by using Taro Yamane formula at 95% confidence interval (Yamane 1967) [15]. As a result, it required a sample size of 398 participants to represent a populations size of 55,424 physicians who were currently practicing medicine nationwide [16].

The survey was carried out by using an online questionnaire via Google Forms, which is an online survey platform operated by Google LLC. The questionnaire was sent to research assistants who are physicians in different hospitals throughout the country. They then sent the link of our online questionnaire to the physicians’ electronic discussion group. The total number of physicians in all groups was 961 members. The survey was on voluntary basis. Only the survey data from those who were willing to answer were sent directly to us.

We newly developed the questionnaire that is suitable and appropriate for the medical situation in Thailand. The content validity of our questionnaire was evaluated by three selected experts comprising one endocrinologist, one preventive medicine specialist, and one community dentistry specialist. The essential and usefulness for achieving the study objective of each single question was evaluated. The accuracy and clarity of the questionnaire were also commented on, and improved by the experts. We adopted the questions with the index of item objective congruence (IOC) larger than 0.5 [17]. The face-to-face cognitive debriefing was then conducted with 11 doctors who attended the residency training program at Ramathibodi hospital, Mahidol University, Bangkok. We further improved the clarity of the questions by interviewing these doctors about their understanding on each individual question. The pilot survey was carried out on 30 physicians with academic and medical backgrounds similar to the targeted participants. The internal consistency of our questionnaire is relatively high with the Cronbach’s alpha coefficients of 0.75.

The final version of the questionnaire consisted of 3 parts. The first part asked the physicians about their demography, job-related information, and medical experience concerning DM. This information was used to examine the potential association of each variable with the attitude of the participants towards DM screening in dental setting. The second part assessed their knowledge of the relationship between DM and PD by using the dichotomous (correct/incorrect) questions. The third part evaluated the attitude of the physicians towards DM screening in a dental clinic, which was assessed by using the 5-point Likert scale. The scale ranges from 1 for the most negative (i.e., very unimportant, very ineffective, or strongly disagree) to 5 for the most positive answers (i.e., very important, very effective, or strongly agree). All parts of the questionnaires are listed in details in Table 1, Table 2 and Table 3, respectively.

Statistical analysis was conducted in 3 separated parts according to the questionnaire: the demographic data, knowledge, and attitude of participants. All parts were analyzed with descriptive statistics based on the frequencies and percentages of answers for each question to demonstrate an overview of the survey data.

In the case of background knowledge, we also used a dichotomous scale (correct/incorrect) and examined the frequencies and percentages of participants answering each question correctly or incorrectly. We divided the participants into 3 groups according to their specialty, i.e., general practitioner (GP), diabetes specialist (DS), and other specialist (OS) [18]. The Pearson’s chi-square or Fisher’s exact test was adopted to evaluate the correlations of correct answers among the physician specialties at the significant level of 0.05.

To assess the attitude of the participating physicians, we calculated the mean score from the 1–5 response scale and defined the favorable outcome as a response scale of 4 and 5, in addition to the descriptive statistics of the frequencies and percentages of each question. All analyses were computed by using PASW statistics for windows version 18.0 (SPSS Inc., Chicago, IL, USA).

## 3. Results

We received a total of 403 completed questionnaires from the participants after excluding five questionnaires from those who were no longer practicing medicine. The response rate was 42.5%. We discuss about this low response rate of our survey in the next section. However, it is noteworthy that 403 random responses were statistically sufficient to represent the entire population of currently practicing physician in Thailand. Demographic distributions of all 403 final participants are summarized in Table 1. We had 205 male and 198 female participants, comprising 51% and 49%, respectively. Sixty-six percent of all respondents were in the age range of 23–30 years old. By categorizing the participants according to their specialty, we had 159 (39.5%) GP and 82 (20.3%) DS, including family medicine practitioners, ophthalmologist, internal medicine practitioners, endocrinologists, and nutritionists. The remaining of 162 (40.2%) participants were OS, such as preventive medicine practitioner, general surgeon, orthopedic surgeon, gynecologist, pediatrician, radiologist, and emergency medicine practitioner.

A majority of participants (65.3%) were working in rural areas, while the rest of 34.7% were in the urban one including Bangkok and perimeter. Most of the participants (63.8%) were working at hospitals affiliated to Ministry of Public Health. The distribution of the working experience was quite consistent with those of the ages in that a majority of the participants (53.4%) had an experience of less than 5 years. The percentage of the participants with 5–10-year working experience was 24.3% and decreased to 22.3% for those having more than 10 years of experiences. Among the total of 403 participants, only 78 (19.4%) participants had ever received the referred case for DM diagnosis from the dentists, 74.4% of which indeed had DM.

Table 2 presents the distribution of knowledge about relationship between DM and PD. It is noteworthy that the descriptive questions in Table 2 all regard ‘correct knowledge’. Therefore, we could simply classify the participants who chose “correct” in their answers as the populations with correct knowledge.

Overall, a majority of the physicians were aware of a relationship between DM and PD. Specifically mentioning, 97.3% of the participants knew about an association between DM and oral diseases (K1 in Table 2). More than 90% of the participants knew that the PD is one of the most important oral diseases of DM patients (93.8%; K2) and poor DM control affects the PD (90.3%; K3). In contrast, the effect of PD on the control of blood sugar level in DM (K4) and the fact that treatment of PD (K5) helps improve glycemic control in DM patients were known by 78.7% and 70.0% of participants, respectively. The distribution of each group based on the specificity of participants is also shown in Table 2. The Fisher exact test was used to examine the association between the correctness of each answer and the specialty of the participating physicians. However, no significant and meaningful association was found.

The questions listed in Table 3 were devoted to exploring the attitude of the physicians toward DM screening in a dental clinic. The distribution of responses, the number of participants with favorable scores, and the average scores of the entire population are summarized in Table 3. The percentage of participants discussed in this section was derived from the favorable score (levels 4 and 5).

According to question A1 in Table 3, most of the respondents (79.2%) thought that a dentist should conduct the DM screening in dental patients. Likewise, 93.1% of the participants suggested a dentist to refer the dental patient with a potential risk of DM to be fully diagnosed by physicians (A2). The screening of DM in dental patients is usually performed by using point of care testing (POCT). Large fraction of the participating physicians (64.5%) considered POCT as an efficient tool to screen DM in the dental clinic (A3). A total of 76.4% and 64% of the respondents thought that it would be beneficial to the patients if a dentist uses POCT to screen DM in dental patients (A4) and monitors DM in the dental patients suffering from DM during clinical visit (A5), respectively. It is also impressive to see that 84.1% of the participating physicians were ready to accept the DM referred cases from the dentists (A6). A substantial number of the respondents (45.7%) also agreed if DM screening were to become routine work at the dental clinic (A7).

We further asked about important issues related to DM screening with finger prick blood test at the dental clinic. The accuracy of the test result was the most concern among all questions in A8 (73.0%). The capability of the dentist who performs the DM screening was another crucial factor about which most of participating physicians were concerned (71.5%). In contrast, increasing the cost for the patients receiving DM screening at the dental clinic was less concerning at 56.1%. Less than half of the participants (43.9%) were concerned about the redundancy of the role between the dentists and the physicians. The answers distributed roughly equally among the scale of not sure, somewhat important, and important (A8.3 and A8.4 in Table 3).

## 4. Discussion

The main aim of this study was to assess the attitude of physicians toward the preliminary DM screening in the dental clinic. Our work is among the very first studies in Thailand. To accomplish this goal, we examined the background knowledge of the participants concerning the association between DM and PD. The results from Table 2 clearly shows that almost all participating physicians knew about the relationship between DM and PD. They knew that the diabetic patients with poor control of blood sugar level are at risk of periodontitis. However, the numbers of physicians knowing the effect of periodontitis on the blood sugar control in diabetic patients, and the effect of periodontal treatment on improving the glycemic control in DM patients, obviously decreased to around 70–80%. Fortunately, the percentages are still considerably high. The similar trend was also seen in the study of Ikimi et al., (2018) [19]. They found 84.3% of Nigerian physicians agreed on the effect of periodontitis on the blood sugar control. Likewise, Tse (2018) [20] studied the awareness of medical practitioners in Hong Kong on the bidirectional relationship between DM and PD and found the percentage falls from 90% for acknowledging the effect of poor DM control on PD to 76% contrariwise. Al-Khabbaz (2011) [21] evaluated the knowledge of medical practitioners in Kuwait and found that 75.4% of participants agreed that DM affects periodontal health. On the other hand, only 40% were aware of the bidirectional association between DM and periodontal health. The above results suggest that the two-way relationship between DM and the PD was inadequately emphasized. The DM-to-periodontitis relationship may be mentioned more frequently than the periodontitis-to-DM relationship in non-dental literature and conferences.

Among all surveys in the attitude section, most of participants agreed that the dentists should refer the dental patients with a potential risk of having DM to the physicians, with the highest percentage being 93.1%. This is in agreement with the second highest percentage where 84.1% of the participating physicians were ready to accept the referred cases from the dentists. It is suggested that the physicians are likely to have a positive attitude toward the primary DM screening in the dental clinics. In fact, almost 80% of the participants considered that dentists should conduct primary DM screening in dental patients.

POCT is a screening service for examining the patient characteristics prior to the dental treatment including the test of the blood sugar level. Baygutalp et al., (2018) [22] conducted a comparative study of blood glucose examining systems using POCT and laboratory methods. They found that the glucometer has sufficient reliability to be used in POCT. Vernillo (2003) [23] proposed an alternative method to examine DM in undiagnosed patients using the cardinal signs of DM and oral complication such as xerostomia or candidiasis. In our study, 64.5% of the physicians thought that the POCT is efficient in screening DM, while more than 70% paid their attention to the accuracy of the DM screening. In addition, a majority of the participants believed that the POCT is beneficial to screen the DM conditions in both undiagnosed and DM patients.

The above results seem to encourage the DM screening to be routine work in the dental clinics. However, Table 3 shows that only half of the participants agreed to have a DM screening as routine work in the dental clinics. The mean score is very neutral at 3.4 (±1.1), where 40% were not sure about their decision. There were only 15% of the physicians who disagreed with the idea of having a primary screening of DM in the dental clinics. This interestingly coincides with the fraction of the participants who were not ready to accept the referred cases from the dental clinics. We examined if this statement were true by individually checking the answers of the participants who disagreed with having a primary DM screening as a routine work. However, there was no statistically significant difference between the group of participants who agreed and those who disagreed with the readiness of accepting referred cases from the dentists.

We further investigated the hypothesis that the knowledge of the bidirectional relationship between DM and the PD may be one of the crucial factors for the physicians in responding to our survey. Table 4 shows the survey results similar to those in Table 3, but from only 246 participants who answered all knowledge questions in Table 2 correctly. By comparing the responses between Table 3 and Table 4, we found only marginally increasing percentages of participants with favorable scores in all questions in Table 4. For example, the percentage of physicians that agreed to have DM screening as routine work in the dental clinic increases from 45.7% for all 403 participants (Table 3) to 51.6% for those 246 participants with all correct answers (Table 4). It is indicated that the knowledge in bidirectional relationship between DM and PD scarcely affects the attitudes of the participants.

It is well established that early identification and appropriate metabolic management of individual with DM can significantly delay the development of most complications [24]. It is also suggested that oral health providers should take an active role in screening certain groups for common medical conditions [25]. Dentists can be one of the important parts in the healthcare team to help reduce the incidence and adverse impact of DM. The collaboration between dentists and physicians must be established to achieve this goal. Our study clearly shows that diabetic screening in a dental clinic is highly acceptable by the physicians. On one hand, the screening is very beneficial for dental patients who are not aware of having diabetic disease. On the other hand, it is also helpful for the diabetic patients if the physicians refer them to the dentists as a high risk of PD.

Despite a clear result of positive attitudes of physicians towards DM screening, our study still has limitations and biases, they being low response rate, an age bias towards young physicians, and an answer bias towards positive attitudes. As mentioned in the previous section, the response rate was 42.5%. It was calculated as the ratio of the number of physicians who answered the questionnaires to the total number of physicians in the electronic discussion group. It is commonly possible that some physicians who were in the electronic discussion group did not actually read the message sent by our research assistants. Some might not even know the existence of the questionnaires in their electronic discussion group. The response rate in this study is likely to be underestimated because it does not mean that the rest of physicians did not want to answer the survey. Various studies that have collected data using Internet surveys also reported the similarly low response rates [26,27,28,29]. We thus believe that the low response rate of our survey does not affect the generalization of the result.

The second limitation and bias with which we are concerned is the age distribution of the participants, which is skewed towards young physicians with ages between 23 and 30 years old. We performed further analysis to check if the age of the respondent affects the result and found no statistically significant correlation between ages and answers in the questionnaires. The reason why we have young physicians as a majority of the respondents (66%) is probably because of the data collecting method, i.e., using the Google form. The physicians who were willing to answer the questionnaires needed to access the link announced in the electronic discussion group and answered all questions via the Google forms. It is possible that our survey would be answered only by those who were already familiar with Internet and electronic devices. This statement is actually in agreement with the results by Olsen et al., (2011) [30]. They found that younger adults with ages between 18 to 28 years old statistically tended to use Internet and technology more frequently than the older group. Finally, it is also possible that the self-reported questionnaires such as in our work may be biased toward one direction of answers over the other. Our online survey was on a volunteer basis. It might be more preferable to those who already agreed with the survey content to answer the questionnaires. However, this is unlikely the case, as the survey responses encompassed the full range of the response scale.

## 5. Conclusions

In summary, we conclude that most of Thai physicians are well aware of the association between DM and PD and acknowledge the bidirectional effect of DM and PD status.

A large majority of physicians agree that DM screening in dental setting is vital and beneficial to dental patients and are ready to accept the patients from the dentists for DM diagnosis. Our study suggests that Thai physicians have a positive attitude towards DM screening in a dental clinic. Dentists should establish more collaboration with physicians to improve treatment DM and PD in the health care system. However, further studies are desirable to explore the awareness and practice guidelines for screening and referring PD patients to the dental clinic. It will build up a better system of health care performed professionally between physicians and dentists.

## Figures and Tables

**Table 1:** General characteristics of participants and referral system.

Characteristic	Total (*n* = 403)
	*n* (%)
**Gender**	
Male	205 (50.9)
Female	198 (49.1)
**Age (years)**	
23–30	266 (66.0)
31–40	76 (18.9)
≥41	61 (15.1)
Mean (sd)	32.1 (8.21)
**Specialty**	
General practice	159 (39.5)
DM specialist	82 (20.3)
Others	162 (40.2)
**Work site**	
Urban	140 (34.7)
Rural	263 (65.3)
**Affiliation**	
Ministry of Public Health	257 (63.8)
Ministry of Education	61 (15.1)
Other government ministry	48 (11.9)
Private sector	37 (9.2)
**Years of practicing**	
<5	215 (53.4)
5–10	98 (24.3)
>10	90 (22.3)
**Received any referred DM case**	
No Yes	325 (80.6) 78 (19.4)
**Percentage of referred cases which are diagnosed with DM**	
≤50%	20 (25.6)
>50%	58 (74.4)

**Table 2:** Basic knowledge concerning association of diabetes mellitus and periodontal disease.

Question	Total	General Practice	DM Specialist	Other Specialist	*p*-Value
	(*n* = 403)	(*n* = 159)	(*n* = 82)	(*n* = 162)	
	*n* (%)	*n* (%)	*n* (%)	*n* (%)	
K1. There is association between diabetes mellitus and oral diseases					0.852 ^1^
Incorrect	11 (2.7)	4 (2.5)	3 (3.7)	4 (2.5)	
Correct ^2^	392 (97.3)	155 (97.5)	79 (96.3)	158 (97.5)	
K2. Periodontal disease (Periodontitis) is one of the most important oral diseases in DM patient					0.625
Incorrect	25 (6.2)	12 (7.5)	5 (6.1)	8 (4.9)	
Correct ^2^	378 (93.8)	147 (92.5)	77 (93.9)	154 (95.1)	
K3. DM patients with poor glycemic control have a risk of developing periodontitis					0.714
Incorrect	39 (9.7)	16 (10.1)	6 (7.3)	17 (10.5)	
Correct ^2^	364 (90.3)	143 (89.9)	76 (92.7)	145 (89.5)	
K4. Periodontal disease may affect the control of blood sugar level in DM patients					0.199
Incorrect	86 (21.3)	41 (25.8)	14 (17.1)	31 (19.1)	
Correct ^2^	317 (78.7)	118 (74.2)	68 (82.9)	131 (80.9)	
K5. Treatment of periodontal disease may help improve glycemic control in DM patients					0.935
Incorrect	121 (30.0)	49 (30.8)	25 (30.5)	47 (29.0)	
Correct ^2^	282 (70.0)	110 (69.2)	57 (69.5)	115 (71.0)	

^1^ *p*-value for dichotomous outcomes based on Fisher-exact test. ^2^ Correct answer. The letter K followed by a number indicates questions in the knowledge part.

**Table 3:** Attitude of medical doctors towards diabetes screening in the dental clinic (*n* = 403).

Topics	Very Unimportant	Somewhat Unimportant	Not Sure	Somewhat Important	Very Important	Favorable Score	Mean (SD)
	*n* (%)	*n* (%)	*n* (%)	*n* (%)	*n* (%)	*n* (%)	
	(1)	(2)	(3)	(4)	(5)	(4, 5)	
A1. Dentist should conduct preliminary DM screening in dental patients	6 (1.5)	15 (3.7)	63 (15.6)	140 (34.7)	179 (44.4)	319 (79.2)	4.2 (0.93)
A2. Dentist should refer dental patients with a potential DM risk to be diagnosed by physician	2 (0.5)	3 (0.7)	23 (5.7)	120 (29.8)	255 (63.3)	375 (93.1)	4.5 (0.68)
**Topics**	**Very ineffective**	**Somewhat ineffective**	**Not sure**	**Somewhat effective**	**Very effective**	**Favorable score**	**Mean (SD)**
	*n* (%)	*n* (%)	*n* (%)	*n* (%)	*n* (%)	*n* (%)	
	(1)	(2)	(3)	(4)	(5)	(4, 5)	
A3. Point of care testing (POCT) is an immediate screening service to measure patient characteristics such as glycemic level with glucose meterHow efficient the DM screening with POCT?	6 (1.5)	26 (6.5)	111 (27.5)	178 (44.2)	82 (20.4)	260 (64.5)	3.8 (0.90)
**Topics**	**Very unbeneficial**	**Somewhat unbeneficial**	**Not sure**	**Somewhat beneficial**	**Very beneficial**	**Favorable score**	**Mean (SD)**
	*n* (%)	*n* (%)	*n* (%)	*n* (%)	*n* (%)	*n* (%)	
	(1)	(2)	(3)	(4)	(5)	(4, 5)	
A4. Will it be beneficial to the patients if a dentist uses POCT to preliminarily screen DM?	7 (1.7)	20 (5.0)	68 (16.9)	153 (38.0)	155 (38.5)	308 (76.4)	4.1 (0.95)
A5. Will it be beneficial to the patients if a dentist monitors DM through POCT for DM patients visiting the dental clinic?	17 (4.2)	43 (10.7)	85 (21.1)	144 (35.7)	114 (28.3)	258 (64.0)	3.7 (1.11)
**Topics**	**Very unready**	**Somewhat unready**	**Not sure**	**Somewhat ready**	**Very ready**	**Favorable score**	**Mean (SD)**
	*n* (%)	*n* (%)	*n* (%)	*n* (%)	*n* (%)	*n* (%)	
	(1)	(2)	(3)	(4)	(5)	(4, 5)	
A6. Readiness in accepting the DM referred case from the dentist	5 (1.2)	7 (1.7)	52 (12.9)	134 (33.3)	205 (50.9)	339 (84.1)	4.3 (0.85)
**Topics**	**Strongly disagree**	**Somewhat disagree**	**Not sure**	**Somewhat agree**	**Strongly agree**	**Favorable score**	**Mean (SD)**
	*n* (%)	*n* (%)	*n* (%)	*n* (%)	*n* (%)	*n* (%)	
	(1)	(2)	(3)	(4)	(5)	(4, 5)	
A7. Would you agree if DM screening becomes routine work at the dental clinic?	20 (5.0)	48 (11.9)	151 (37.5)	115 (28.5)	69 (17.1)	184 (45.7)	3.4 (1.06)
**Topics**	**Very unimportant**	**Somewhat unimportant**	**Not sure**	**Somewhat important**	**Very important**	**Favorable score**	**Mean (SD)**
	*n* (%)	*n* (%)	*n* (%)	*n* (%)	*n* (%)	*n* (%)	
	(1)	(2)	(3)	(4)	(5)	(4, 5)	
A8. If a dentist needs to perform DM screening in dental patients using finger prick blood test at dental clinic, how important do you think it is for each of the following issues?							
A8.1 Accuracy of the test result	3 (0.7)	20 (5.0)	86 (21.3)	148 (36.7)	146 (36.2)	294 (73.0)	4.0 (0.92)
A8.2 Ability of a dentist to perform DM screening	7 (1.7)	25 (6.2)	83 (20.6)	164 (40.7)	124 (30.8)	288 (71.5)	3.9 (0.96)
A8.3 Increasing cost of DM screening at the dental clinic and definite diagnosis by the physician	12 (3.0)	40 (9.9)	125 (31.0)	136 (33.8)	90 (22.3)	226 (56.1)	3.6 (1.03)
A8.4 Role redundancy between dentists and physicians	32 (7.9)	58 (14.4)	136 (33.8)	99 (24.6)	78 (19.4)	177 (43.9)	3.3 (1.17)

The letter A followed by a number indicates questions in the attitude part.

**Table 4:** Attitude of medical doctor who provide correct answer in the knowledge session towards diabetes screening in dental clinic (*n* = 246).

Topics	Very Unimportant	Somewhat Unimportant	Not Sure	Somewhat Important	Very Important	Favorable Score	Mean (SD)
	*n* (%)	*n* (%)	*n* (%)	*n* (%)	*n* (%)	*n* (%)	
	(1)	(2)	(3)	(4)	(5)	(4, 5)	
A1. Dentist should conduct preliminary DM screening in dental patients	1 (0.4)	5 (2.0)	33 (13.4)	87 (35.4)	120 (48.8)	207 (84.2)	4.3 (0.81)
A2. Dentist should refer dental patients with a potential DM risk to be diagnosed by physician	0 (0.0)	1 (0.4)	6 (2.4)	66 (26.8)	173 (70.3)	239 (97.2)	4.7 (0.54)
**Topics**	**Very ineffective**	**Somewhat ineffective**	**Not sure**	**Somewhat effective**	**Very effective**	**Favorable score**	**Mean (SD)**
	*n* (%)	*n* (%)	*n* (%)	*n* (%)	*n* (%)	*n* (%)	
	(1)	(2)	(3)	(4)	(5)	(4, 5)	
A3. Point of care testing (POCT) is an immediate screening service to measure patient characteristics such as glycemic level with glucose meterHow efficient the DM screening with POCT?	3 (1.2)	8 (3.3)	57 (23.2)	117 (47.6)	61 (24.8)	178 (72.4)	3.9 (0.85)
**Topics**	**Very unbeneficial**	**Somewhat unbeneficial**	**Not sure**	**Somewhat beneficial**	**Very beneficial**	**Favorable score**	**Mean (SD)**
	*n* (%)	*n* (%)	*n* (%)	*n* (%)	*n* (%)	*n* (%)	
	(1)	(2)	(3)	(4)	(5)	(4, 5)	
A4. Will it be beneficial to the patients if a dentist uses POCT to preliminarily screen DM?	2 (0.8)	10 (4.1)	29 (11.8)	102 (41.5)	103 (41.9)	205 (83.3)	4.2 (0.86)
A5. Will it be beneficial to the patients if a dentist monitors DM through POCT for DM patients visiting the dental clinic?	6 (2.4)	26 (10.6)	44 (17.9)	89 (36.2)	81 (32.9)	170 (69.1)	3.9 (1.07)
**Topics**	**Very unready**	**Somewhat unready**	**Not sure**	**Somewhat ready**	**Very ready**	**Favorable score**	**Mean (SD)**
	*n* (%)	*n* (%)	*n* (%)	*n* (%)	*n* (%)	*n* (%)	
	(1)	(2)	(3)	(4)	(5)	(4, 5)	
A6. Readiness in accepting the DM referred case from the dentist	4 (1.6)	3 (1.2)	23 (9.4)	82 (33.3)	134 (54.5)	216 (87.8)	4.4 (0.83)
**Topics**	**Strongly disagree**	**Somewhat disagree**	**Not sure**	**Somewhat agree**	**Strongly agree**	**Favorable score**	**Mean (SD)**
	*n* (%)	*n* (%)	*n* (%)	*n* (%)	*n* (%)	*n* (%)	
	(1)	(2)	(3)	(4)	(5)	(4, 5)	
A7. Would you agree if DM screening become routine work at the dental clinic?	10 (4.1)	26 (10.6)	83 (33.7)	76 (30.9)	51 (20.7)	127 (51.6)	3.5 (1.06)
**Topics**	**Very** **unimportant**	**Somewhat unimportant**	**Not sure**	**Somewhat important**	**Very important**	**Favorable score**	**Mean (SD)**
	*n* (%)	*n* (%)	*n* (%)	*n* (%)	*n* (%)	*n* (%)	
	(1)	(2)	(3)	(4)	(5)	(4, 5)	
A8. If a dentist needs to perform DM screening in dental patients using finger prick blood test at dental clinic, how important do you think it is for each of the following issues?							
A8.1 Accuracy of the test result	1 (0.4)	8 (3.3)	54 (22.0)	94 (38.2)	89 (36.2)	183 (74.4)	4.1 (0.86)
A8.2 Ability of a dentist to perform DM screening	3 (1.2)	10 (4.1)	53 (21.5)	104 (42.3)	76 (30.9)	180 (73.2)	4.0 (0.89)
A8.3 Increasing cost of DM screening at the dental clinic and definite diagnosis by the physician	9 (3.7)	18 (7.3)	83 (33.7)	87 (35.4)	49 (19.9)	136 (55.3)	3.6 (1.00)
A8.4 Role redundancy between dentists and physicians	21 (8.5)	29 (11.8)	89 (36.2)	62 (25.2)	45 (18.3)	107 (43.5)	3.3 (1.16)

The letter A followed by a number indicates questions in the attitude part.

## Data Availability

The data presented in this study are available on request from the corresponding author. The data are not publicly available due to ethical issue.
